# Dynamic integration and excision of filamentous phage XacF1 in *Xanthomonas citri* pv. *citri*, the causative agent of citrus canker disease

**DOI:** 10.1002/2211-5463.12312

**Published:** 2017-09-19

**Authors:** Abdelmonim A. Ahmad, Makoto Kawabe, Ahmed Askora, Takeru Kawasaki, Makoto Fujie, Takashi Yamada

**Affiliations:** ^1^ Department of Molecular Biotechnology Graduate School of Advanced Science of Matter Hiroshima University Higashi‐Hiroshima Japan; ^2^ Department of Plant Pathology Faculty of Agriculture Minia University El‐minia Egypt; ^3^ Floral and Nursery Plants Research Unit US National Arboretum USDA/ARS, BARC‐West Beltsville MD USA; ^4^ Department of Microbiology Faculty of Science Zagazig University Zagazig Egypt

**Keywords:** biocontrol, citrus canker, filamentous phage, integration mechanism, XerC/D

## Abstract

Inovirus XacF1 (7325 nucleotides) is integrated into the genome of *Xanthomonas citri* pv. *citri* (*Xcc*) strains at the host *dif* site (*att*B) by the host XerC/D recombination system. The XacF1 *att*P sequence is located within the coding region of ORF12, a possible phage regulator. After integration, this open reading frame (ORF) is split into two pieces on the host genome. We examined dynamic integration/excision of XacF1 in *Xcc* strain MAFF 301080 and found that the integration started at 4 h postinfection (p.i.) and peaked at 12 h p.i. Thereafter, the ratio of integrated to free forms remained constant, suggesting equilibrium of integration and excision of XacF1 in the host genome. However, the integrated state became very unstable following a 5′‐deletion of ORF12 in XacF1, suggesting that ORF12 plays a key role in the integration cycle of XacF1 in *Xcc* strains.

**Abbreviations**
ACC: Asian citrus canker
EPS: exopolysaccharide
MM: minimal medium
NA: nutrient agar
NB: nutrient broth
ORF: open reading frame
p.i.: postinfection
*Xcc*: *Xanthomonas citri* pv. *citri*

Asian citrus canker (ACC), caused by *Xanthomonas citri* pv. *citri*,* Xcc* (syn., *Xanthomona saxonopodis* pv. *citri* and *Xanthomonas campestris* pv. *citri*), is one of the most serious diseases in citrus‐producing areas of the tropical and subtropical countries 1, 2. Typical symptoms of ACC include raised corky lesions on leaves, stems, and fruits, including general tree decline, defoliation, twigs dieback, blemished fruit, and premature fruit drop in severely infected trees 3. The causative agent *Xcc* encodes and expresses many genes involved in the pathogenesis and virulence 4. Some relevant genes identified in *Xcc* are the ones coding for cell motility, plant cell wall‐degrading enzymes, protein secretion systems, toxins, exopolysaccharide (EPS) synthesis enzymes, etc. 5. Recently, a filamentous bacteriophage (inovirus) XacF1 infecting *Xcc* strains was isolated and characterized 6. This phage encodes 13 open reading frames (ORF) and its genome is 7325 nucleotides in size (Fig. S1). In contrast to lytic phages, infection with filamentous phages does not cause host cell lysis, but frequently establishes a persistent association between the host and phage, producing and releasing phage particles from the growing and dividing host cells. After infection by XacF1, the bacterial host cells showed several cultural and physiological changes including lower levels of EPS production, reduced motility, slower growth rate, and a dramatic reduction in the virulence 6. This virulence‐lowering effect of XacF1 infection suggests that XacF1 could be used as a biocontrol agent against citrus canker pathogens. However, it was also shown that XacF1 was frequently integrated in the host genome, potentially limiting the effect of XacF1 as a biocontrol agent if the phage needs to be present in the free‐replicating state. This integration occurred at the host *dif* site (*att*B) like *Vibrio cholerae* phage CTXφ by the host XerC/D recombination system 6. Some inoviruses, once integrated into the host genome via the host XerC/D, can be excised in the reverse direction through the same recombination system to be new extrachromosomal phage copies 7. Because phage infection effects on the host (such as the virulence‐lowering effect) may change depending on the phage states, namely a free‐replicating state or a prophage state 8, we are interested to characterize the dynamics of XacF1 infection and integration in the *Xcc* host cells.

## Materials and methods

### Bacterial culture and phage infection

*Xanthomonas citri* pv. *citri* MAFF 301080 was obtained from the National Institute of Agrobiological Sciences, Japan. The cells were grown on nutrient agar (NA) medium (Difco, Cockeysville, MD, USA) at 28 °C. For the preparation of bacterial suspension, *Xcc* cells were cultured for 24 h at 28 °C with shaking at 220 r.p.m. in nutrient broth (NB) medium (BBL; Becton Dickinson and Co., Cockeysville, MD). Bacteriophage XacF1 was isolated as described previously 6. Phage infection was performed as follows: When the cultures reached 0.2 unit of OD_600_, XacF1 was added at a multiplicity of infection (moi) of 0.001–1.0. After further growth for 12–24 h, the cells were spread on NA plates with appropriate dilutions. Single colonies were picked and purified for further studies. For phage preparation, the cells were removed by centrifugation with an R12A2 rotor in a Hitachi himac CR21E centrifuge at 8000 ***g*** for 15 min at 4 °C. The supernatant was passed through a 0.45‐μm pore membrane filter followed by precipitation of the phage particles in the presence of 0.5 M NaCl and 5% polyethylene glycol 6000 (Kanto Chemical Co., Tokyo, Japan).

### XacF1 integration assay

To detect integrated forms of XacF1 phage in the host genome after infection, we conducted PCR experiments with total DNA isolated from XacF1‐infected MAFF 301080 cells at various times postinfection (p.i.) DNA was isolated with a Genome DNA kit (Macherey‐Nagel GmbH & Co. KG, Duren, Germany) and adjusted exactly to 100 μg·mL^−1^ for quantitative comparison. For the detection of integrated forms, primer sets of 5′ GAT CGA ATA TGG ATG CAC GGT GTA G 3′ (forward) and 5′ TGC CTG GTG CTC GAG CTG CAG TGC AGA TGG C 3′ (reverse), corresponding to the host sequence immediately upstream of *att*B and a part of XacF1 genome, were used, respectively (Fig. S2). PCR products were separated by agarose gel electrophoresis, and the DNA sequence was confirmed for the flanking region (*att*R). For free‐replicating forms, XacF1 sequences 5′ ACG GGT TTT TCT TGA CAG AGA GGA CAG TGA AAA C 3′ (forward), corresponding to XacF1 DNA positions 6887–6920, and 5′ ATG ACG CTA GAC ACC TAC GAT CGC GTA GAC 3′ (reverse), corresponding to XacF1 DNA positions 6401–6430, were used to span the *att*P sequence. For quantitative comparison, *Xcc* 16S rDNA sequence was amplified with primers 5′ AAC GCG AAG AAC CTT ACC TGG TCT 3′ (forward) and 5′ TGC GGG ACT TAA CCC AAC ATC TCA 3′ (reverse; accession no. CP006857.1). Twenty‐five rounds of PCR were performed under standard conditions in a LifePro Thermal Cycler (Bioer Technology Co., Ltd., Binjiang, China).

### Knockout of ORF12 and construction of ΔXacF1‐mutant phages

A complete coding region of ORF12 was deleted from XacF1 DNA by PCR using a forward primer 5′ GAC GGG TTT TTC TTG ACA GAG AGG ACA GTG 3′ (corresponding to XacF1 DNA positions 6886–6916) and a reverse primer 5′ CAC AGG AAG CGG ACT GGA CAA TGA CGC T 3′ (corresponding to XacF1 DNA positions 6423–6450; Fig. S1). Another mutant construct of XacF1 that lacks a 5′ major portion of ORF12 but retains *att*P was formed by PCR with a forward primer 5′ GAC GGG TTT TTC TTG ACA GAG AGG ACA GTG 3′ (corresponding to XacF1 DNA positions 6886–6916) and a reverse primer 5′ CCC TCT CGC CGG TAG CCG TTA CCC ACC 3′ (corresponding to XacF1 DNA positions 6522–6548). The PCR products were extracted and purified from the agarose gel after electrophoretic separation and circularized with T4 DNA ligase after phosphorylation with polynucleotide kinase. The resulting DNA was introduced into the cells of *Xcc* MAFF 301080 (without any XacF1 prophage and susceptible to XacF1) by electroporation 9. Transformed cells were cultivated to obtain RF phage DNA. The mutant phage constructs were confirmed by direct sequencing.

### Preparation of *Xcc* competent cells

Electrocompetent cells of *Xcc* were prepared as previously described 10 with some modifications. Briefly, an overnight subculture of *Xcc* cells (MAFF 301080) was used to seed 500 mL of NB medium. The bacterial cells were grown in NB medium with shaking (140 r.p.m.) at 28 °C to a density of OD_600_ = 0.6, harvested by centrifugation (4000 ***g***), for 10 min, at 4 °C, washed twice under the same condition with 500 mL of distilled sterile water (0 °C), and resuspended in 500 μL of sterile 10% glycerol at 0 °C. Samples (50 μL aliquots) were kept in liquid nitrogen until use.

### Electroporation

Electrocompetent cells (50 μL) were slowly thawed in an ice bath and mixed with 1–2 μL of transforming DNA solution. The mixture was immediately transferred to a chilled electroporation cuvette (0 °C). Electroporation was performed using a Gene Pulser Xcell (Bio‐Rad, Hercules, CA, USA) with a 2‐mm cell at 2.5 kV, according to the manufacturer's manual. Following the pulse, 1 mL of NB medium was immediately added to the cells and the bacterial suspension was transferred to a polypropylene tube. After a room temperature incubation of 1 h, the tube was shaken at 140 r.p.m. for 4 h at 28 °C. Then, the bacterial cells were subjected to plaque assay with MAFF 301080 cells as the host. Single plaques were isolated and phage‐containing cells were cultivated to obtain the replicative‐form phage DNA (RF).

### Biological and physiological assays

To compare biological and physiological changes that occurred in the *Xcc* cells infected with wild‐type or mutant XacF1 phages, several assays were conducted as follows: EPS production was determined according to Guo *et al*. 11. Briefly, bacterial cells (*Xcc* strain MAFF 301080) were grown in NB supplemented with 2% glucose for 24 h at 28 °C with shaking at 200 r.p.m. A 10 mL portion of the culture was collected, and the cells were removed by centrifugation (5000 ***g*** for 20 min). The supernatant was mixed with three volumes of 99% ethanol and the mixture was kept at 4 °C for 30 min. The dry weights of EPS were measured. Twitching motility was assayed as described previously 6. Overnight bacterial cultures in NB were centrifuged at 8000 ***g*** for 2 min at 4 °C, washed twice with ddH_2_O, and resuspended in ddH_2_O. Two microlitre of the suspension was spotted on minimal medium (MM) plates 12. Plates were incubated at 28 °C, and the morphology of the colony edge was observed under a culture microscope at 4–10× magnification (an Olympus CKX41, Tokyo, Japan). Virulence assay was performed with lemon leaves (immature fully expanded lemon leaves) as described before 6. Needle‐prick inoculation was performed by pricking the leaves and droplets (10 μL) of bacterial suspensions (10^8^ colony‐forming units·mL^−1^) were added to each inoculation site. Leaves were incubated in a growth chamber at 28 °C (12‐h light and 12‐h dark) for 4 weeks.

## Results

### Effects of ORF12 mutation on XacF1 integration into the host genome

**Figure 1:**
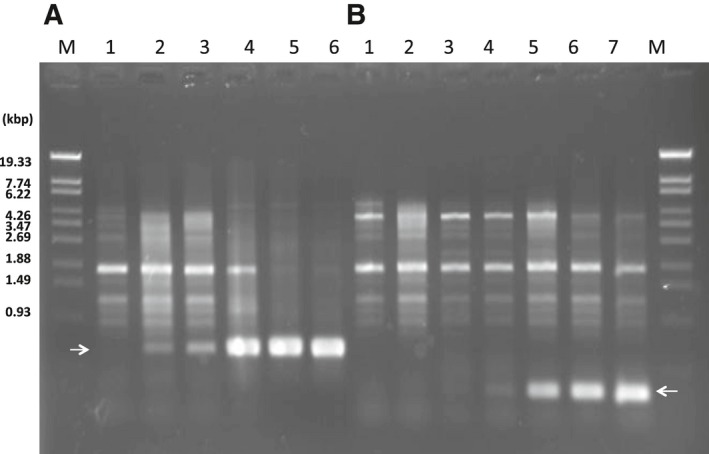
Detection of integrated forms of XacF1 after infection in *Xcc* MAFF 301080 as a host. Cells were infected with wild‐type XacF1 (A) or with a ∆XacF1′ mutant that lacked a 5′ major portion of ORF12, but retained *att*P (B). Arrows indicate bands corresponding to integrated forms. Lanes: M, molecular marker (lambda‐*Sty*I fragments); 1, 2 h p.i.; 2, 4 h p.i.; 3, 6 h p.i.; 4, 12 h p.i.; 5, 24 h p.i.; 6, 48 h p.i.; 7, 72 h p.i.

XacF1 integration into the host genome was directly detected by PCR experiments. When wild‐type XacF1 was infected with *Xcc* strain MAFF 301080, the integrated form was detected (a band of ca. 550 bp in size) as early as 4 h p.i., and increased its integrated amounts to a peak level at 12 h p.i. (Fig. 1A). In contrast, a deletion mutant of XacF1 (∆XacF1) that lacks an entire region of ORF12 (including *att*P) could infect the host cells with forming plaques, but did not give any consistent integration bands in PCR (data not shown). This result agreed with the idea that XacF1 integrates into the host *dif* site by homologous recombination at the *att*P site. Meanwhile, another mutant of XacF1 (∆XacF1′) that lacked a 5′ major portion of ORF12 but retained the *att*P site became ssDNA phages and its titer on MAFF 301080 as a host was approximately the same (or even higher) as that of wild‐type XacF1. ∆XacF1′ showed a somewhat delayed integration (detected as a PCR product of ca. 270 bp in size) as shown in Fig. 1B. In this case, the integration signal was detected at 12 h p.i. and its peak was reached at 48–72 h p.i. This delayed integration could be caused by the function of ORF12 itself or by changing sequences flanking *att*P in the XacF1 genome.

### Effects of ORF12 mutation on the stability of integrated forms of XacF1 in the host genome

Some inoviruses, once integrated into the host genome via the host XerC/D, can be excised in the reverse direction through the same recombination system to be new extrachromosomal phage copies 7, 8. This integration and excision should be dynamically regulated by some mechanism in the host cells. To evaluate the possible involvement of ORF12 in this mechanism, we compared stability of XacF1 prophage between the cells infected with wild‐type XacF1 and with ∆XacF1′.

**Figure 2:**
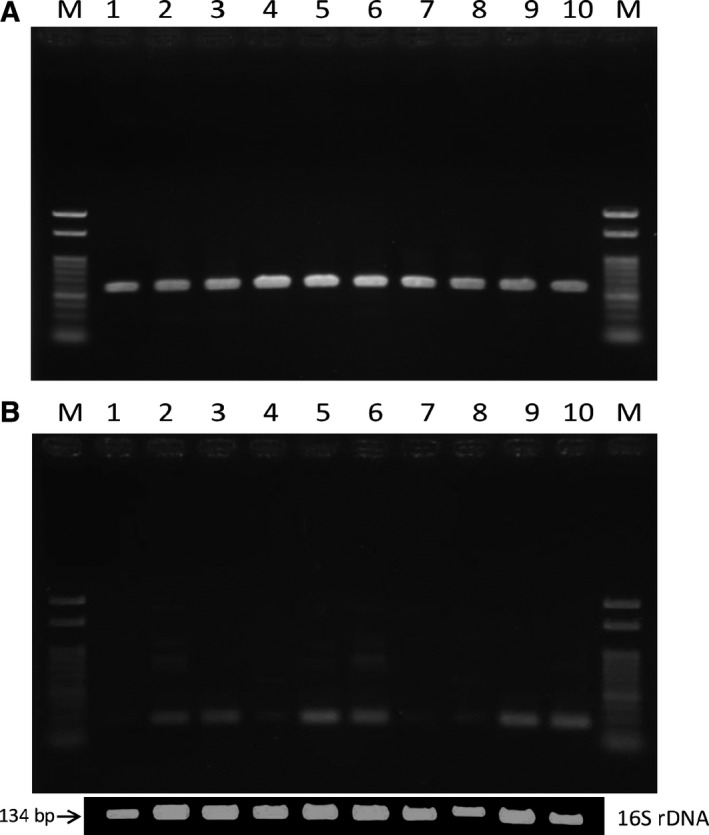
Stability of integrated forms of XacF1 in *Xcc* MAFF 301080 cells. (A) A single colony containing a XacF1 prophage was picked and cultivated in NB for 48 h at 28 °C. The cells were then spread onto NA plates, and colonies were formed after three days. Ten single colonies were picked and subjected to PCR for the detection of XacF1‐integrated forms. (B) The same experiments were performed with ∆XacF1′‐integrated cells. Lanes: M, molecular size marker (100‐bp ladder), 1–10, independent single colonies. For comparison, 16S rDNA fragments were also amplified in the same way (Materials and methods). There are two identical rRNA operons in the *Xcc* genome 18.

A single colony containing a XacF1 prophage was picked and cultivated in NB for 48 h at 28 °C. The cells were then spread onto NA plates and colonies were formed. After 48‐h incubation at 28 °C, 10 single colonies were picked and subjected to PCR for the detection of XacF1‐integrated forms. As shown in Fig. 2A, XacF1 prophage states were very stable and all 10 colonies gave almost equivalent amounts of integration signals. However, ∆XacF1′‐prophage states were relatively unstable and the prophage signals varied significantly among the colonies; a few showed only very faint signals (Fig. 2B). These results suggest that ∆XacF1′, where almost the entire region of ORF12 was deleted, integrates into the host cells relatively inefficiently and once integrated, its forms are maintained unstably and easily excised to be extrachromosomal. In other words, ORF12 possibly works as an integration‐enhancing and integration‐stabilizing element in XacF1.

### Effects of ORF12 mutation in XacF1 on the host virulence

XacF1 infection caused drastic changes to host cells including lowering levels of EPS production, reducing motility, slowing growth rate, and dramatically reducing the virulence 6. As shown above, XacF1 integrates into the host genome as early as 4 h p.i., and the prophage states are maintained stably thereafter. Here, the XacF1 mutants offered an opportunity to compare the effects of XacF1 infection on the host between the free forms and prophage forms.

**Figure 3:**
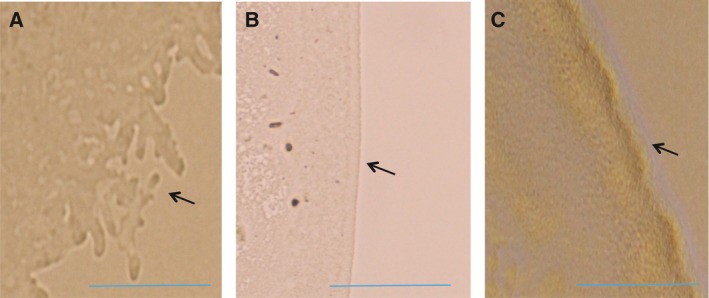
Twitching motility of XacF1‐infected bacterial cells. Two microlitre of the cell suspension (*Xcc* MAFF 301080) was spotted on MM plates. Plates were incubated at 28 °C, and the morphology of the colony edge was observed under a culture microscope (4–10× magnification). (A) Uninfected cells, (B) XacF1‐infected cells, and (C) ∆XacF1′‐infected cells. Bar represents 0.5 mm.

When twitching motility was observed on assay plates, the colony margin of uninfected cells showed highly irregular shapes, indicating proficient twitching motility (Fig. 3A). This twitching state was indistinguishable from that of cells infected with ∆XacF1. Meanwhile, the colony edge of XacF1‐integrated cells was smooth (Fig. 3B), suggesting a decrease or loss of twitching motility. ∆XacF1′‐infected cells showed reduced size, and the morphology of the colony edge was observed under increased magnification (Fig. 3C).

Exopolysaccharide production in XacF1‐integrated cells showed a significant reduction (~ 0.6 ± 0.3 mg/10 mL culture, *n* = 3) compared to uninfected cells (6.7 ± 2.0 mg/10 mL culture, *n* = 3). Meanwhile, EPS levels were almost the same in the culture of cells infected with ∆XacF1, but were found to be significantly reduced in the ∆XacF1′‐infected cells (1.2 ± 1.0 mg/10 mL culture, *n* = 3).

**Figure 4:**
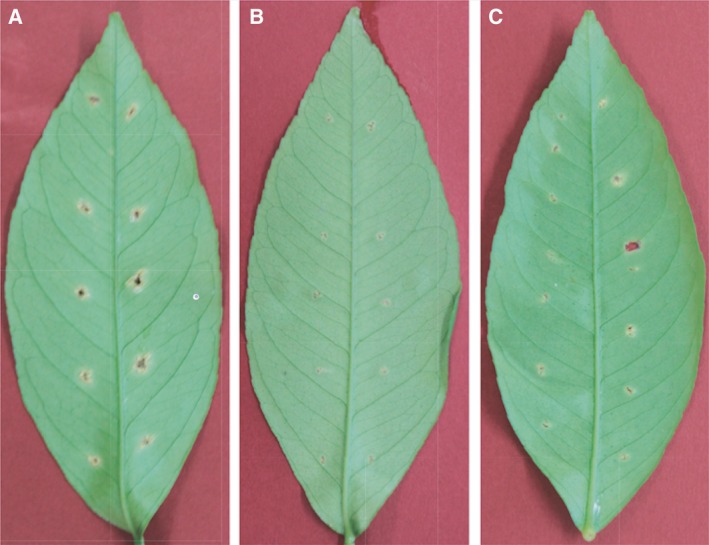
Canker symptoms developed on lemon leaves 1 week p.i by the needle‐pricking method. Leaves were inoculated with uninfected cells (A), with XacF1‐infected cells (B), and with ∆XacF1′‐infected cells (C). Each leaf was inoculated with cells of 10 independent single colonies.

*Xanthomonas citri* pv. *citri* strain MAFF 301080 virulence was observed after infection with XacF1 containing ORF12 mutations. In the virulence assay using the pricking method with lemon leaves, wild‐type cells of strain MAFF 301080 caused infection symptoms after 3–4 days postinfection (d.p.i.) and formed clear canker symptoms 1 week after inoculation (Fig. 4A). On the other hand, the symptoms of XacF1‐infected cells were very weak and no mature canker could be seen up to 4 weeks p.i., except for marginal lesions formed around pricking sites (Fig. 4B). When lemon leaves were pricked with cells infected with ∆XacF1, canker symptoms were obvious, appearing almost the same as leaves inoculated with uninfected cells. Meanwhile, the symptoms of ∆XacF1′‐infected cells were variable (Fig. 4C); some lesions were significantly large (almost the same as those formed by uninfected cells) and others were very small but with canker symptoms 6. These results are summarized in Table 1.

**Table 1:** Effects of ORF12 mutation in XacF1 on the host properties

	Twitching motility	EPS production^a^	Virulence^b^
No infection	+	+	+
XacF1‐lysogenic	−	−	−
∆XacF1	+	+	+
∆XacF1′	−	±	±

a ‘+, > 5 mg/10 mL culture; ±, 1–5 mg/10 mL culture; ‘−, < 1 mg/10 mL culture.

b ‘+, showing obvious canker symptoms; ±, showing canker symptoms, but very small in some cases; ‘−, no canker symptoms.

## Discussion

Inoviruses coexist with their host cells, meaning that infection by these phages can influence host bacterial phenotypes in various ways. In pathogenic bacteria of either animals or plants, virulence is frequently affected by phage infection. For example, infection of *X. campestris* pv*. oryzae* NP5850 by the filamentous phages Xf and Xf2 enhanced virulence, possibly because of overproduction of extracellular polysaccharides (EPS) by the phage‐infected bacterial cells 13. In contrast with the virulence‐enhancing effects of ϕRSS1 14, phage‐mediated loss of virulence was also reported in *Ralstonia solanacearum*. *Ralstonia solanacearum* completely lost virulence through infection with two other filamentous phages ϕRSM1 and ϕRSM3 12. Many virulence factors were found to be significantly reduced in ϕRSM‐infected cells. These opposing effects of different filamentous phages on *R. solanacearum* virulence make it an ideal study model system for understanding the effect of filamentous phage on their hosts. In the same way with φRSM, XacF1 phage shows many significant effects on the physiological features of *Xcc*‐infected cells. These effects include slower growth rate; lower EPS production; and reduced cells' swimming, swarming, and twitching motility; and dramatic reduction in virulence 6. This loss of virulence effect of XacF1 infection could be explained by the reduction in the growth rate, EPS production, and cell motility. It is also known that XacF1 can integrate into the host genome by site‐specific recombination by the host XerC/D system 6. This has raised a question of how those phage effects vary depending on the phage states, namely a free‐replicating state or a prophage state. In this study, it was found that XacF1 starts to integrate into the host genome as early as 4 h p.i. and increased integrated forms to a peak level of 12 h p.i. (Fig. 1). However, in the form of ∆XacF1 where ORF12 was completely deleted, no integration occurred. This is because the *att*P was deleted in the XacF1 genome. As shown in Table 1, cells infected with this form (a free‐replicating state) were not drastically different in physiological properties compared with noninfected cells. In contrast, in cells infected with ∆XacF1′, where *att*P was intact and a 5′ major portion of ORF12 was deleted, integration occurred but was inefficient and delayed compared with wild‐type XacF1 (Fig. 1). Once integrated, the prophage state of ∆XacF1′ was unstable and easily released from the host genome as shown in Fig. 2B. These results strongly suggest that ORF12 plays a key role to regulate (facilitate and stabilize) the prophage state of XacF1. Because the ∆XacF1′‐infected cells showed very unstable features such as lowered twitching motility, lowered EPS production, and variable virulence (Figs 3 and 4, and Table 1), the virulence‐lowering effect of XacF1 infection might be caused by stable integration of XacF1 into the host genome. In this sense, ORF12 containing *att*P and a regulatory function on the XacF1 genome may play an important role in regulating not only integration dynamics, but also virulence states of the host cells.

It is worth noting that the ORF12 region of XacF1 corresponds to ‘the primary immunity determinant’ of *Xcc* phage Cf 15. Cf is the first filamentous phage found to infect *Xcc* strains 16 and was shown to be integrated into the host chromosome 17. Cheng *et al*. 15 showed that mutations in the primary immunity determinant of Cf resulted in integration defect. Therefore, our observation that the integrated state became very unstable following a 5′‐deletion of ORF12 in XacF1, suggesting that ORF12 plays a key role in the integration cycle of XacF1 in *Xcc* strains, agrees with the Cf results.

## Author contributions

TY and AAA conceived the studies and designed the experiments that were performed by AAA, MK, and AA. TK and MF contributed to the construction of deletion mutants and plant assay, respectively. TY wrote the manuscript together with AAA, TK, and MF.

## Supporting information


**Fig. S1.** Genomic organization of bacteriophage XacF1.
**Fig. S2.** A model for integration/excision of XacF1 in *Xanthomonas citri*.
