# Supplementary material for: Dynamic integration and excision of filamentous phage XacF1 in Xanthomonas citri pv. citri, the causative agent of citrus canker disease
Source: FEBS Open Bio. 2017 Sep 19;7(11):1715–21. doi: 10.1002/2211-5463.12312 (PMC5666396; doi:10.1002/2211-5463.12312)
Supplement: Supplementary file 1 — Fig. S1. Genomic organization of bacteriophage XacF1. Fig. S2. A model for integration/excision of XacF1 in Xanthomonas citri. [file FEB4-7-1715-s001.pdf]

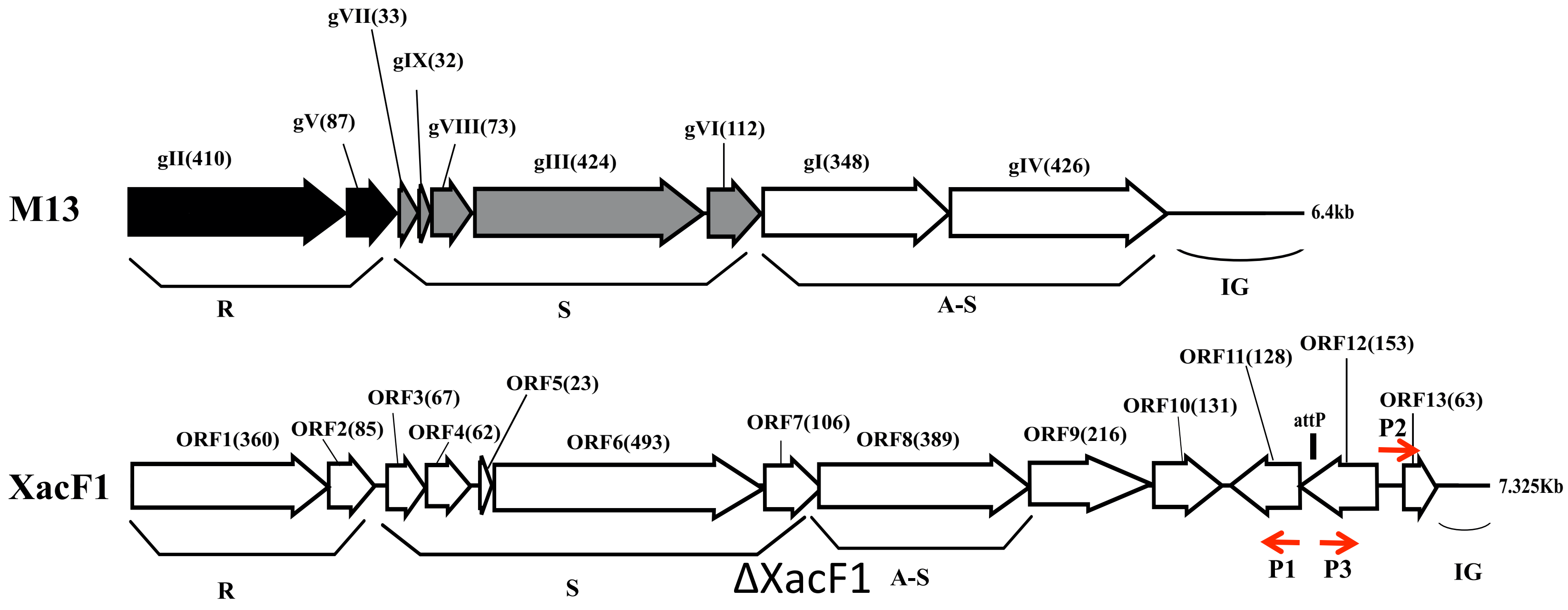

Supplementary Fig. S1. Genomic organization of bacteriophage XacF1. A linear genomic map (7,325 nt) is compared with that of *E. coli* phage M13. Arrows oriented in the direction of transcription represent ORFs or genes. The functional mudules for replication (R), structure (S), and assembly-secretion (A-S) are indicated. Red arrows show positions and directions of PCR primers used in ΔXacF1 formation (P1 and P2 for ΔXacF1 and P1 and P3 for ΔXacF1'.

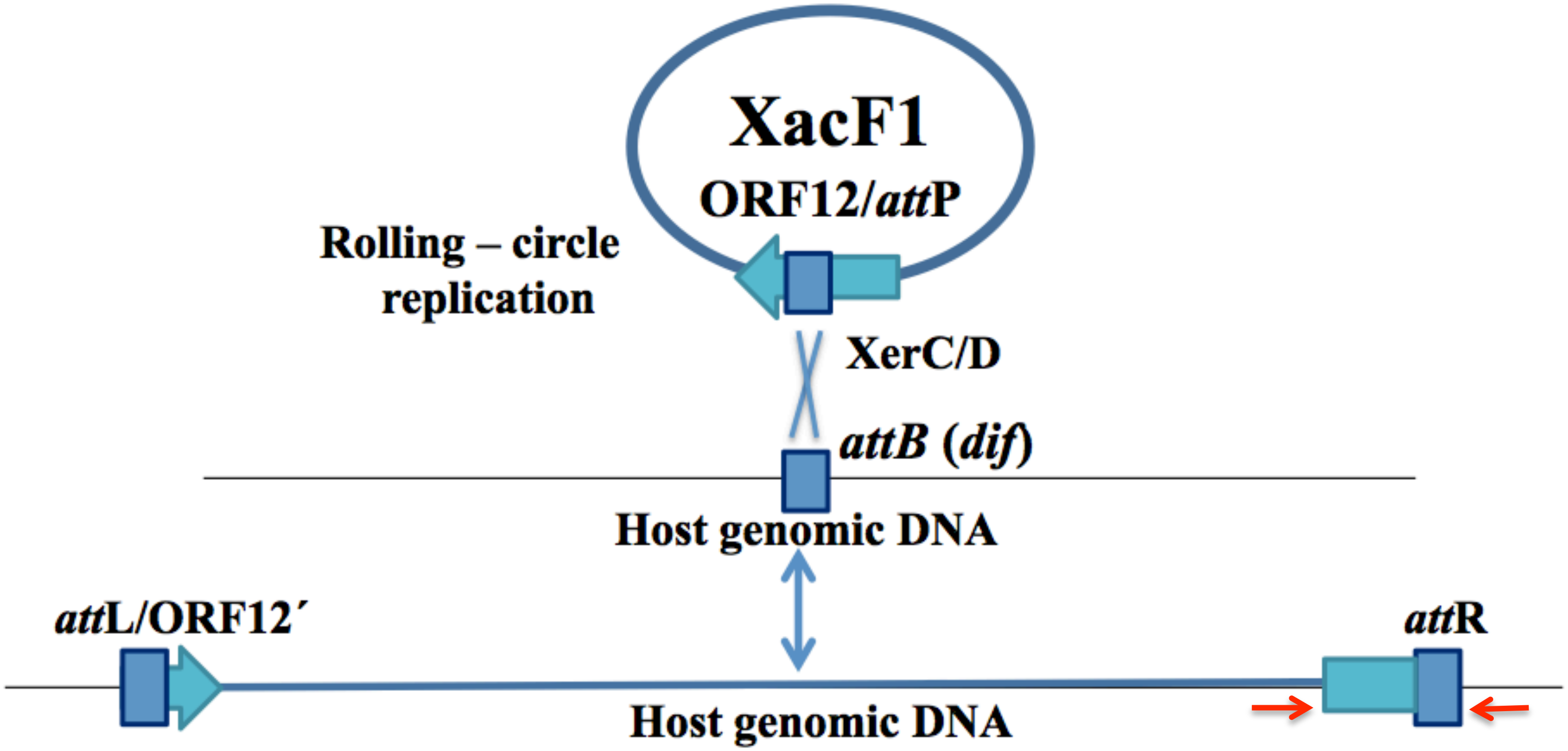

Supplementary Fig. S2. A model for integration/excision of XacF1 in *Xanthomonas citri*. XacF1 integration occurrs by recombination between *att*P within ORF12 and *dif* (*att*B) on the host genome by XerC/D recombinases. After integration ORF12 is split into two portions (ORF12') beside *att*L and *att*R. Red arrows indicate PCR primers to detect integrated forms.
